# Serum kisspeptin levels mainly depend on ovarian expression of *Kiss1* mRNA in female rats

**DOI:** 10.3389/fphys.2022.998446

**Published:** 2022-11-15

**Authors:** Ahreum Kwon, Ji Young Eom, Woo Jung Lee, Han Saem Choi, Kyungchul Song, Junghwan Suh, Hyun Wook Chae, Ho-Seong Kim

**Affiliations:** Department of Pediatrics, Severance Children’s Hospital, Endocrine Research Institute, College of Medicine Yonsei University, Seoul, South Korea

**Keywords:** kisspeptins, Kiss1, ovary, hypothalamus, puberty, female, rats

## Abstract

The hypothalamic kisspeptin/KISS1 receptor system is essential for puberty onset and reproductive development. Although serum kisspeptin might be associated with puberty, its levels, according to developmental stage, and its origin still remain unclear. This study evaluated the changes in serum kisspeptin levels during puberty and the corresponding *Kiss1* mRNA and protein expression in various organs of female rats to identify the source of serum kisspeptin. Tissues from several organs, including the ovaries and anteroventral periventricular nucleus (AVPV) and arcuate nucleus (ARC) in the hypothalamus, were obtained for assessing *Kiss1* mRNA and protein expressions. Serum kisspeptin levels progressively increased with developmental stages until the peripubertal stage. The ovaries showed the highest *Kiss1* expression among the organs examined. Next, we explored the changes in serum kisspeptin levels and hypothalamic *Kiss1* expression in ovariectomized and estradiol-treated ovariectomized rats. Serum kisspeptin levels decreased regardless of estradiol treatment; *Kiss1* expression was enhanced by ovariectomy and estradiol treatment in the ARC, while it was decreased by ovariectomy and enhanced by estradiol in the AVPV, suggesting that serum kisspeptin may be associated with pubertal development and mainly depended on ovarian *Kiss1* expression. Thus, serum kisspeptin levels are associated with puberty and may serve as a downstream marker of ovarian reproductive function.

## 1 Introduction

Puberty is a highly orchestrated and regulated process that occurs by activating the hypothalamic–pituitary–gonadal (HPG) axis. The HPG axis is a complex biological system that is not yet fully characterized. Kisspeptin, produced by *KISS1*, acts as a gatekeeper for puberty onset *via* its cognate receptor GPR54 (also known as the KISS1 receptor [KISS1R]) (Ohtaki et al., 2001; Seminara et al., 2003). The kisspeptin/KISS1R system has been established as a regulator of puberty based on studies showing that inactivating and activating mutations in either *KISS1* or *KISS1R* were associated with hypogonadotropic hypogonadism and precocious puberty (de Roux et al., 2003; Seminara et al., 2003; Topaloglu et al., 2012; Novaira et al., 2014). In addition, *KISS1* is expressed within the hypothalamus, especially in the anteroventral periventricular nucleus (AVPV) and arcuate nucleus (ARC) (Smith et al., 2005; Clarkson and Herbison, 2006), and its expression increases as puberty progresses (Clarkson and Herbison, 2006; Semaan and Kauffman, 2015). In animal studies, central infusion of a kisspeptin antagonist suppresses gonadotropin-releasing hormone (GnRH) pulses and reduces luteinizing hormone (LH) pulse frequency (Adachi et al., 2007; Li et al., 2009; Roseweir et al., 2009), while central administration of kisspeptin induces precocious activation of the HPG axis and results in precocious puberty (Navarro et al., 2004). Therefore, the kisspeptin/KISS1R system is an upstream regulator of GnRH release and has been proposed to play an important role in the onset of puberty.

Since hypothalamic kisspeptin affects puberty onset, serum kisspeptin has been suggested as an attractive marker for puberty onset. Evaluating the serum kisspeptin level, according to pubertal stage, may help determine whether it can be used as a biomarker for puberty. To date, several studies (Vries et al., 2009; Pita et al., 2011; Rhie et al., 2011; Jayasena et al., 2014; Xue et al., 2020) have shown conflicting results regarding serum kisspeptin levels during puberty. Serum kisspeptin levels increase with age and peak around puberty (Jayasena et al., 2014); they are significantly higher in girls with central precocious puberty than pre-pubertal girls of the same age (Vries et al., 2009; Rhie et al., 2011). In contrast, there were no differences in serum kisspeptin levels between pre-pubertal and pubertal groups (Pita et al., 2011) or between a central precocious pubertal and normal group (Xue et al., 2020). Therefore, a detailed evaluation of the change in serum kisspeptin levels according to developmental stage is urgently needed.

Identifying the origin of serum kisspeptin might elucidate the association between serum kisspeptin levels and puberty. *KISS1* is widely distributed not only in the hypothalamus but also other organs, such as the pituitary gland, placenta, kidneys, pancreas, adrenal gland, adipose tissue, testes, and ovaries (Lee et al., 1996; Terao et al., 2004; Gaytán et al., 2009). Thus, it is not clear whether changes in *Kiss1* expression in the hypothalamus reflect changes in serum kisspeptin, or whether these changes are brought about by some other organ. Emerging evidence has indicated potential physiological roles of extra-hypothalamic kisspeptins in modulating puberty and the reproductive system (Laoharatchatathanin et al., 2015; Uenoyama et al., 2016). In particular, ovaries express *Kiss1* (Terao et al., 2004; Castellano et al., 2006; Laoharatchatathanin et al., 2015), which seems to be controlled by LH (Castellano et al., 2006). Furthermore, local administration of a high dose kisspeptin antagonist to an ovary exerts a negative influence on puberty onset (Ricu et al., 2012). Therefore, the ovary is also suspected to be a candidate organ for the main source of serum kisspeptin. Investigating the association between serum kisspeptin levels and changes in *Kiss1* expression in various organs, including the hypothalamus and ovary at different developmental stages, will help determine which organ expressing *Kiss1* is the main source of serum kisspeptin during puberty. This analysis might help in understanding the biological significance of serum kisspeptin levels during puberty.

In this study, we explored changes in serum kisspeptin levels in female rats from the neonatal stage to puberty. We also evaluated *Kiss1* mRNA and protein expression in several organs during development to explore the source of serum kisspeptin. In addition, we investigated serum kisspeptin levels and hypothalamic *Kiss1* mRNA and protein expression in ovariectomized (OVX) rats, with and without estradiol replacement, to explore the role of the ovaries and hypothalamus in regulating serum kisspeptin levels.

## 2 Materials and methods

### 2.1 Animals and study design

The experimental procedures used in this study were approved by the Institutional Animal Care and Use Committee of Yonsei University College of Medicine (approval number 2016-0046) and conducted in accordance with the Guide for the Care and Use of Laboratory Animals (1996 [7th ed.] Washington, DC: National Research Council, National Academies Press). To ensure that all the female rats used were of the same conditions, timed-pregnant Sprague–Dawley rats (17th day of pregnancy, *n* = 8) were purchased from Japan SLC, Inc., (Shizuoka, Japan). Each pregnant rat bore six to eight female littermates, and a total of 56 littermates were used in this study. The current study was designed to minimize the number of animals used. Female littermate rats born before 1,000 h were considered to be 1 day old. The animals were maintained under standard conditions of a 12:12 light: dark cycle (lights on at 0800 h) and a temperature of 22°C. The rats were weaned on day 21 and housed, three per cage, with free access to pellet food and tap water. Body weights were checked every morning at 1,000 h. To confirm the completion of puberty, vaginal opening (VO), which was defined by the vagina being pink, wrinkled, and completely canalized [25], was also checked every morning at 1,000 h. The developmental stages of the female rats were defined as follows: neonate (days 1–7), infant (days 8–21), peripubertal (days 22–31), and pubertal completion (day of VO) (Ojeda and Urbanski, 1988). To evaluate serum kisspeptin patterns at differential development stages, serum samples were obtained at onset and half-way through each developmental stage; that is, on day 4 (P4, middle of neonate stage), day 8 (P8, onset of infancy), day 14 (P14, mid-infancy), day 23 (P23, onset of peripuberty), day 27 (P27, mid-peripuberty), and the day of VO (completion of puberty). Each day, samples were taken from four to eight female rats. To evaluate *Kiss1* mRNA and protein expression in the hypothalamus, pituitary gland, ovaries, uterus, adrenal glands, and pancreatic tissues, tissue samples from these organs were obtained at the same time points. The rats were euthanized by decapitation between 1,000 h and 1,100 h. The hypothalamus was removed according to the rat brain atlas (Khazipov et al., 2015), using a micro knife to make a 2 mm-deep horizontal cut that began 1 mm away (in the anterior direction) from the optic chiasm and continued to the posterior borders of the mammillary bodies and the hypothalamic fissures. The AVPV and ARC were compartmentalized according to the rat brain atlas (Khazipov et al., 2015). The anterior and posterior ends of the AVPV tissue sections were approximately 0.84 mm and 0.60 mm posterior to the bregma, respectively. The anterior and posterior ends of the ARC tissues were approximately 1.80 and 4.08 mm posterior to the bregma, respectively.

Next, to evaluate the role of ovarian *Kiss1* expression on serum kisspeptin levels, we measured serum kisspeptin levels and hypothalamic *Kiss1* mRNA and protein expression in OVX rats. Rats were subjected to ovariectomies at P14, and serum samples and hypothalamic tissues were obtained on P23, P27, and P34. Because OVX rats had a delayed pubertal onset, samples were obtained at P34 (mean day of VO in intact female rats), instead of the day of VO. We also measured serum kisspeptin levels and *Kiss1* mRNA and protein expression in the hypothalamus in OVX rats after estradiol replacement to explore the regulatory effect of estradiol on serum kisspeptin levels. We subcutaneously administered estradiol (E-8875, Sigma Chemical Co., St. Louis, MO) at a dose of 25 μg/kg/day from P14 to the day of VO. Serum samples and hypothalamic tissues were obtained on P23, P27, and the day of VO.

### 2.2 Measuring serum kisspeptin levels

Serum samples were collected by immediate centrifugation for 15 min at 1,000 × g at 4°C and stored at −70°C before determining serum kisspeptin levels. Serum kisspeptin levels were measured using a highly sensitive enzyme-linked immunosorbent assay kit (E-EL-R2530, Elabscience Biotech, Wuhan, China) with a detection range of 78.13–5,000 pg/ml. Serum kisspeptin concentration was measured by diluting the blood of rats to one-fiftieth.

### 2.3 RNA extraction and reverse transcription polymerase chain reaction (RT-PCR) analysis

To determine *Kiss1* mRNA expression in tissues at each developmental stage, RNA was extracted and analyzed by RT-PCR. Tissues from several rat organs were removed immediately following decapitation, frozen in liquid nitrogen, and stored at −80°C until being processed for mRNA analyses. Total RNA was isolated using the Trizol reagent (Invitrogen, Carlsbad, CA, United States). A total of 2 µg RNA was synthesized using LaboPass cDNA kit (Cosmo Gentech, Seoul, South Korea) according to the manufacturer’s instructions. Primer sequences used for RT-PCR were obtained from the published sequence of *Kiss1* (GenBank accession number AY196983.1; Table 1), and *β-actin* was used as the reference gene. PCR was carried out at an initial denaturation cycle at 95°C for 5 min, followed by a variable number of amplification cycles defined by denaturation at 94°C for 40 s, annealing at 55°C for 40 s, and 30 cycles of extension at 72°C for 50 s. A final extension cycle of 72°C for 10 min (Takara, Japan) was also included. PCR products were separated on 1.5% agarose gels and visualized by ethidium bromide staining.

**TABLE 1:** RT-PCR primer pair sequences.

Gene	Forward primer	Reverse primer
*β-actin*	5′-TGT​CAC​CAA​CTG​GGA​CGA​TA-3′	5′-TCT​CAG​CTG​TGG​TGG​TGA​AG-3′
*Kiss1*	5′-ACT​CGT​TAA​TGC​CTG​GCA​AA-3′	5′-AGG​CCA​AAG​GAG​TTC​CAG​TT-3′

### 2.4 mRNA analyses by reverse transcription quantitative real-time PCR (RT-qPCR)

The *Kiss1* mRNA levels in representative samples from female rats in different developmental stages were quantified by RT-qPCR. RT-qPCR was performed with 20 µl of PCR amplification reaction mixture containing 900 ng of complementary DNA, hydrolysis probe (*Kiss1*: Rn00710914_m1, *β-actin*: Rn00667869_m1), and TaqMan Universal PCR Master Mix (Applied Biosystems, Foster City, CA, United States). Amplification was performed in duplicate with the following thermocycling profile: 50°C for 2 min, 95°C for 10 min, and 40 cycles at 95°C for 15 s and 60°C for 1 min. A StepOnePlus instrument (Applied Biosystems) was used for the reactions. The relative expression of mRNA was calculated using the 2^−ΔΔCt^ method (Rao et al., 2013). The reference samples were arbitrarily taken from each organ from 4-day-old female rats. RT-qPCR analyses were performed in triplicate.

### 2.5 Western immunoblotting analysis

To determine the protein expression levels of Kiss1, we performed western immunoblotting analysis. Frozen hypothalamus, ovary, pituitary gland, adrenal gland, and uterus tissues were homogenized and lysed on ice in RIPA lysis buffer (WSE-7420, ATTO, Tokyo, Japan.) Protein extracts were centrifuged at 17,000 × *g* for 30 min. Then, the protein concentrations were determined using the BCA protein assay (Applygen Technologies Inc., Beijing, China). Equal amounts of proteins (150 µg) were boiled for 5 min at 100°C with loading buffer (containing 8% *β*-mercaptoethanol) for denaturation. The amount of the protein used as a reference gene was 20 μg, and the experiment was repeated. The denatured proteins were loaded on a 12.5% sodium dodecyl sulfate-polyacrylamide gel electrophoresis (SDS-PAGE) gel for 1 h. The proteins were separated by SDS-PAGE and transferred to a 0.25-µm polyvinylidene fluoride membrane for 30 min. The membranes were blocked with phosphate-buffered saline (PBS) containing 5% skim milk for 30 min at room temperature (as around 20–22°C). Then, the membranes were incubated separately with primary antibodies against kisspeptin (1:500, catalog number GTX130503, GeneTex, Irvine, California, USA) and *β*-actin (1:3000, catalog number #4970, Cell Signaling Technology, Danvers, Massachusetts, United States) at 4°C overnight. The polyclonal kisspeptin antibody is of IgG isotype and reacts with human, mouse, and rat kisspeptin. After washing thrice with PBS containing 0.2% Tween 20 (PBS-T) for 10 min, the membranes were incubated with a horseradish peroxidase-conjugated secondary goat anti-rabbit antibody (1:5000) for 30 min at room temperature, followed by four washes in PBS-T for 10 min. Then, the membranes were exposed to enhanced chemiluminescence. Blots were exposed to medical X-ray film.

### 2.6 Statistical analyses

Quantitative RNA data are presented as the mean ± standard error of the mean. One-way ANOVA followed by Tukey’s test was performed to compare changes in serum kisspeptin levels at different developmental stages. To compare changes in serum kisspeptin levels in OVX and estradiol-treated OVX female rats at different developmental stages, two-way (age and OVX/estradiol-treated OVX) ANOVA was performed. In addition, if age-by-OVX/estradiol-treated OVX interaction effects were significant with *p*-value < 0.05, then an independent *t*-test was performed to confirm the treatment effect on each group by post-hoc analysis. The Bonferroni multiple comparison test was used for multiple comparisons. All analyses were performed using SAS software (version 9.2; SAS Inc., Cary, NC, United States). *p* values < 0.05 were considered to reflect statistically significant differences.

## 3 Results

### 3.1 Developmental profile of serum kisspeptin levels in intact female rats

**FIGURE 1:**
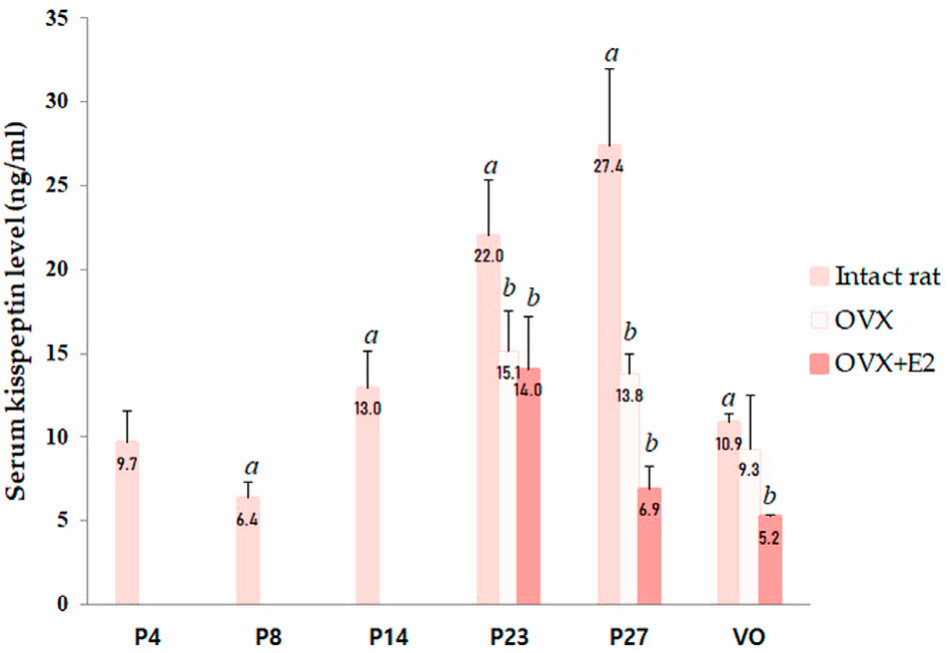
Changes in serum kisspeptin levels at different developmental stages. Serum kisspeptin levels in intact female rats; changes in serum kisspeptin levels in ovariectomized (OVX) and estradiol-treated OVX female rats (OVX + E2) were measured. Specimens were collected on days 4, 8, 14, 23, and 27, and on the day of vaginal opening (VO) in intact female rats. Ovariectomy was conducted on day 14; specimens were collected on days 23, 27, and on the day of vaginal opening (VO) or day 34 in OVX and OVX-estradiol treated rats. Each group had four to eight animals. Data are presented as the mean ± standard error of mean. ^*a*^Significant difference (*p* < 0.05) in treatments just before each stage; ^*b*^Significant difference (*p* < 0.05) in levels compared to those in intact female rats.

Serum kisspeptin levels were analyzed at different developmental stages, and each group contained four to eight animals. Serum kisspeptin levels were moderate during the neonatal period, slightly decreased in early infancy, and significantly increased during successive developmental stages (compared to just before stages, all *p* < 0.05), peaking at peripuberty (P27) (Figure 1). However, the levels began to decrease at the completion of puberty (*p* < 0.001) (Figure 1).

### 3.2 Kiss1 mRNA and protein expressions in various organs in intact female rats according to the developmental stage

**FIGURE 2:**
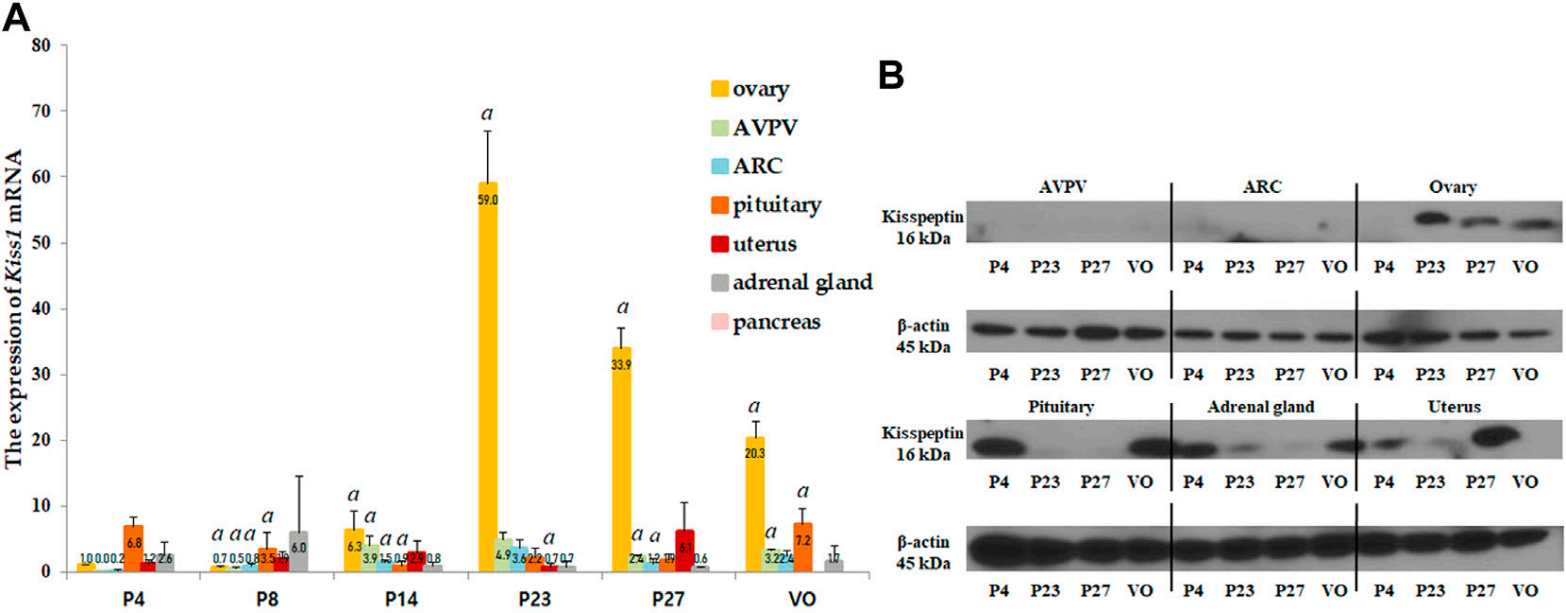
**(A)** Developmental profiles of *Kiss1* mRNA expression in several organs, such as the anteroventral periventricular nucleus (AVPV) and arcuate nucleus (ARC) in the hypothalamus, and the pituitary gland, ovaries, uterus, adrenal glands, and pancreas. Specimens were collected on days 4, 8, 14, 23, and 27, and on the day of vaginal opening (VO). *Kiss1* mRNA expression were quantified by RT-qPCR in triplicate. Each value was quantified on the basis of the value of *Kiss1* mRNA-expression in the ovary on day 4. **(B)** Protein expression levels of Kiss1 were quantified by western immunoblotting in triplicate. Each group had four to eight animals. Data are presented as the mean ± standard error of mean. ^*a*^Significant difference (*p* < 0.05) in treatments just before each stage.

**FIGURE 3:**
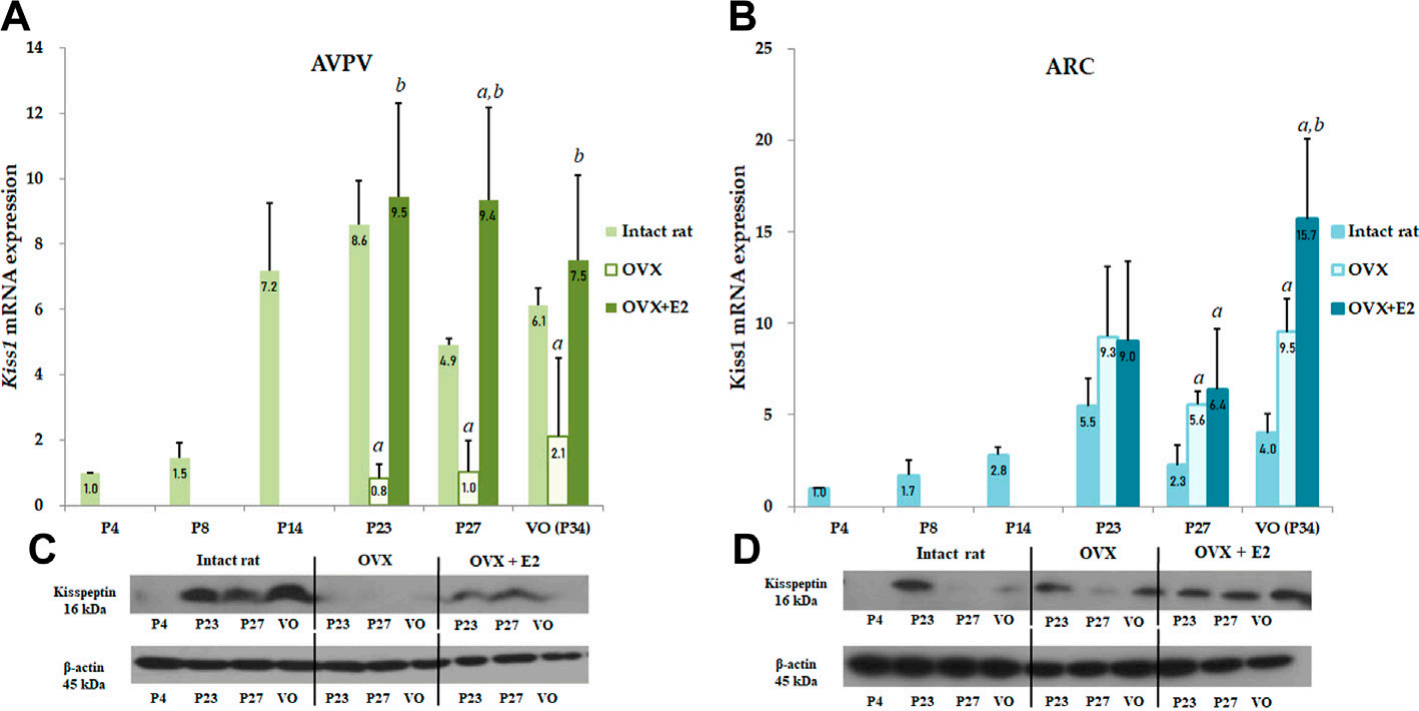
Developmental profiles of *Kiss1* mRNA and protein expression in the anteroventral periventricular nucleus (AVPV) and arcuate nucleus (ARC) in intact female, ovariectomized (OVX), and estradiol-treated OVX rats (OVX + E2) from the neonatal stage to the onset of puberty. Ovariectomy was conducted on day 14, and specimens were collected on days 23, 27, and on the day of vaginal opening (VO) or day 34. Each group contained four to eight animals **(A)** *Kiss1* mRNA expression of the AVPV was quantified by RT-qPCR. Each value was quantified on the basis of the value of *Kiss1* mRNA expression in the AVPV on day 4. **(B)** *Kiss1* mRNA expression of the ARC was quantified by RT-qPCR. Each value was quantified on the basis of the value of *Kiss1* mRNA expression in the ARC on day 4. **(C)** Kiss1 protein levels in the AVPV were quantified by western immunoblotting. **(D)** Kiss1 protein levels in the ARC were quantified by western immunoblotting. Each group contained four to eight animals. RT-qPCR analyses and western immunoblotting analyses were performed in triplicate. Data are presented as the mean ± standard error of the mean. ^*a*^Significant difference (*p* < 0.05) in levels compared to those in intact female rats; ^*b*^Significant difference (*p* < 0.05) in levels compared to those in OVX rats.

*Kiss1* mRNA and protein expression were analyzed in several organs, including the AVPV and ARC in the hypothalamus, pituitary gland, ovaries, uterus, adrenal glands, and pancreas. *Kiss1* mRNA in each organ was expressed relative to the *Kiss1* mRNA in the ovaries of 4-day-old female rats. The ovaries expressed the highest levels of *Kiss1* mRNA near the onset of puberty, and the pituitary gland expressed the second highest levels (Figure 2A). *Kiss1* mRNA was barely expressed in the pancreas (Figure 2A). In the ovaries, the *Kiss1* mRNA levels were lowest from the neonatal to infant periods but significantly rapidly increased at the peripubertal stage (*p* < 0.001), peaking on P23, and then gradually decreasing until puberty completed (Figure 2A). Changes in *Kiss1* mRNA expression in the ovaries corresponded to changes in serum kisspeptin levels, although they were one developmental step ahead of the corresponding serum kisspeptin levels. That is, changes in mRNA expression corresponding to the developmental stage were observed prior to the changes observed in serum levels. *Kiss1* mRNA expression in the AVPV was low from the neonatal period until early infancy, then increased during the peripubertal stages and peaked at P23 (compared to just before the stage at P14, *p* < 0.05, Figures 2A, 3A). Similarly, *Kiss1* mRNA levels in the ARC were moderate during the neonatal period, gradually increased during developmental stages until P23, and slightly decreased at P27 and VO (compared to just before the stage at P14, *p* < 0.05, Figures 2A, 3B). Although *Kiss1* mRNA expression in the pituitary gland was persistent and increased at VO (compared to just before the stage, both *p* < 0.01), no specific pattern was observed during developmental stages (Figure 2A).

We then performed western immunoblotting analysis to determine changes in the protein expression of Kiss1 in various organs at P4, P23, P27, and VO. Similar to the *Kiss1* mRNA expression pattern, the ovaries expressed the highest levels of Kiss1 protein at P23 and P27 (Figure 2B).

### 3.3 Changes in serum kisspeptin levels in OVX and estradiol-treated OVX (OVX + E2) female rats at different developmental stages

We performed ovariectomy and measured the serum kisspeptin level in OVX female rats to elucidate the effects of the ovaries on changes in serum kisspeptin levels. In addition, the serum kisspeptin levels in OVX female rats were compared with those in estradiol-treated OVX female rats to determine whether the change in serum kisspeptin level was due to the ovaries or estradiol. Since there was a significant interaction effect between developmental stage and OVX/estradiol-treated OVX for serum kisspeptin (*p* < 0.001), we confirmed the change in serum kisspeptin according to OVX or estradiol-treated OVX at each developmental stage. The serum kisspeptin levels in OVX female rats were significantly lower than those in intact female rats at P23 (*p* < 0.01) and P27 (*p* < 0.001); after adjusting the age, the serum kisspeptin levels in intact female rats were higher than those in OVX female rats (*β* = −8.595, SE = 2.175, *p* < 0.001). The serum kisspeptin levels in OVX female rats did not recover after estradiol treatment (compared with those in intact female rats; P23, *p* < 0.01; P27 and VO, *p* < 0.001); after adjusting the age, the serum kisspeptin levels in estradiol-treated OVX rats were significantly lower than those in intact female rats (*β* = −6.339, SE = 1.235, *p* < 0.001) (Figure 1).

### 3.4 Changes in hypothalamic Kiss1 mRNA and protein expression in OVX and estradiol-treated OVX female rats

To determine the effect of *Kiss1* expression in the AVPV and ARC on serum kisspeptin levels, *Kiss1* mRNA and protein expression in the AVPV and ARC were measured in both OVX and estradiol-treated OVX rats and compared to the serum kisspeptin pattern. In the AVPV, there was a significant interaction effect between developmental stage and OVX/estradiol-treated OVX (*p* < 0.05). We confirmed the change in *Kiss1* mRNA and protein expression in the AVPV according to OVX/estradiol-treated OVX at developmental stages. *Kiss1* mRNA expression in the AVPV was significantly lower in OVX female rats than in intact rats at all developmental stages (P23 and P27, *p* < 0.001; P34, *p* < 0.01), even after adjusting the age (*β* = −5.384, SE = 1.207, *p* < 0.001) (Figure 3A). However, these levels normalized after estradiol treatment, implying that *Kiss1* mRNA expression was increased by estradiol (compared with those in OVX rats, P23, *p* < 0.01; P27 and VO, *p* < 0.001; VO) (Figure 3A). After adjusting the age, *Kiss1* mRNA expression in AVPV was recovered and increased after estradiol treatment in OVX female rats (*β* = 2.482, SE = 0.749, *p* = 0.002). In contrast, *Kiss1* mRNA expression in the ARC was increased in OVX rats compared to that in intact female rats (P27 and VO, *p* < 0.01) (Figure 3B), and was also statistically significant after adjusting the age (*β* = 4.215, SE = 0.809, *p* < 0.001). After estradiol administration, as well as after adjusting the age (*β* = 2.991, SE = 0.630, *p* < 0.001), *Kiss1* mRNA expression also increased more than that in intact female rats (P27, *p* < 0.05; VO, *p* < 0.01). Furthermore, *Kiss1* mRNA expression in estradiol-treated OVX rats increased even more than that in OVX rats at VO (*p* < 0.05); however, it was not statistically significant after adjusting the age (*β* = 2.258, SE = 1.676, *p* = 0.192) (Figure 3B). Kiss1 protein expression measured by western immunoblotting analysis showed a pattern similar to *Kiss1* mRNA expression in the AVPV and ARC (Figures 3C,D).

## 4 Discussion

This study evaluated patterns in serum kisspeptin levels in different developmental stages and investigated the main source of serum kisspeptin in female rats. Serum kisspeptin level increased progressively in accordance with the developmental stage until the peripuberty stage, suggesting that it may be an associated marker of puberty. In addition, we evaluated *Kiss1* expression in various organs at similar stages and demonstrated that the pattern of *Kiss1* expression in the ovary, AVPV, and ARC was similar to that of serum kisspeptin. Especially, the ovaries expressed the highest levels of *Kiss1* mRNA and Kiss1 protein near the onset of puberty. Because the highest expression of *Kiss1* mRNA and Kiss1 protein during development was observed in the ovary, we evaluated the changes in serum kisspeptin level, and the *Kiss1* mRNA and protein expression in the AVPV and ARC in OVX and estradiol-treated OVX rats. Serum kisspeptin level decreased in OVX rats independent of estradiol treatment, unlike the changes in *Kiss1* mRNA expression in the AVPV and ARC. These results suggest that the ovaries are the main source of serum kisspeptin, and an increase in serum kisspeptin levels along with the progression of puberty is not related to *Kiss1* expression in the AVPV and ARC.

Just as kisspeptin in the AVPV and ARC in the hypothalamus control pubertal development (Ohtaki et al., 2001; Seminara et al., 2003), peripheral serum kisspeptin might also have significant implications in pubertal development (Vries et al., 2009; Rhie et al., 2011; Young et al., 2013; Jayasena et al., 2014; Decourt et al., 2016; Xue et al., 2020). However, due to conflicting results (Pita et al., 2011; Xue et al., 2020), studies on the patterns of serum kisspeptin levels in successive developmental stages are required to clarify the pattern of serum kisspeptin. To the best of our knowledge, this is the first study to evaluate the patterns of serum kisspeptin levels at different developmental stages in female rats. We found that serum kisspeptin levels increased progressively during the sequential developmental stages until the peri-pubertal stage, suggesting that serum kisspeptin may reflect puberty or serve as a marker associated with puberty.

However, it remains unclear whether the increase in serum kisspeptin levels during successive developmental stages reflects the changes in hypothalamic *Kiss1* expression that occur at puberty onset. A recent report showed that serum kisspeptin levels were elevated in girls compared to boys, suggesting that the higher levels may be due to greater hypothalamic kisspeptin signaling in girls than in boys (Jayasena et al., 2014). However, since kisspeptin is expressed in several other organs (Lee et al., 1996; Terao et al., 2004; Gaytán et al., 2009), these organs may also be candidates that control the serum kisspeptin levels during developmental stages. Kanasaki et al. (2013) suggested that only local kisspeptin, which is produced within the hypothalamic neuron, may exert its stimulatory effect on GnRH *via* a paracrine mechanism and that circulating kisspeptin may originate from other peripheral organs. Little effort has been directed toward determining the source of serum kisspeptin, and only a limited number of studies have been performed to analyze the significance of *Kiss1* expression in various organs according to the developmental stage. Therefore, to better identify the origin of serum kisspeptin, we evaluated changes in *Kiss1* mRNA and protein expression in several organs known to express *Kiss1* according to the developmental stage in intact female rats. In this study, while the other organs sparsely expressed *Kiss1* mRNA, *Kiss1* mRNA and/or did not show significant patterns depending on the development stage, expression prominently increased with the progression of puberty in the ovary and the AVPV and ARC, similar to the changes in serum kisspeptin levels.

To test this possibility more specifically, we investigated serum kisspeptin levels and *Kiss1* expression in the AVPV and ARC in both OVX and estradiol-treated OVX rats. Serum kisspeptin levels decreased significantly in OVX rats, suggesting that the ovaries are the main organs that contribute to serum kisspeptin or that estrogen exclusion, due to ovariectomy, decreased *Kiss1* expression in the AVPV, thereby decreasing serum kisspeptin levels. Previous studies (Roa et al., 2006; Smith et al., 2006; Kauffman et al., 2007; Takase et al., 2009) have shown that *Kiss1* expression decreased after ovariectomy and increased with estradiol treatment in the AVPV, whereas an opposing trend was observed in the ARC. These results led to the hypothesis that estradiol may play a positive-feedback role in the AVPV in regards to kisspeptin, whereas in the ARC, it may serve a negative-feedback role (Smith et al., 2005; Roa et al., 2006). In this study, changes in *Kiss1* mRNA and protein expression in the AVPV in OVX and estradiol-treated OVX rats were consistent with the results of previous studies (Smith et al., 2005; Kauffman et al., 2007; Takase et al., 2009). However, the decrease in the serum kisspeptin level in OVX rats, which was similar to the reduced *Kiss1* expression in the AVPV, was not restored even after estradiol treatment, which was different from *Kiss1* expression in the AVPV in estradiol-treated OVX rats. In addition, although the change in *Kiss1* expression in the ARC in OVX and estradiol-treated OVX rats was not consistent with the finding of previous studies, *Kiss1* expression increased in OVX and estradiol-treated OVX rats. These findings suggest that *Kiss1* expression in the ovaries, rather than in the hypothalamus mainly regulates serum kisspeptin level. Furthermore, the serum kisspeptin level in OVX rats was not restored to the level observed in intact female rats even after estradiol treatment. In a previous study on goose ovaries, the serum kisspeptin level was negatively correlated with serum estradiol level (Hua et al., 2014). Therefore, the findings of this study confirmed that the serum kisspeptin level is not regulated by ovarian estradiol.

In the present study, although there was no characteristic change according to the developmental stage, *Kiss1* mRNA expression was high in the pituitary gland. In addition to the expression in hypothalamus, expression of both *Kiss1* and *Kiss1r* in the pituitary has been reported in several species (Kauffman et al., 2007; Richard et al., 2008; Witham et al., 2013; Ikeda et al., 2017). Ikeda et al. (2017) examined the expression profile of kisspeptin in the mouse pituitary at all stages, and *Kiss1* mRNA was detected in the rostroventral portion and the dorsocaudal portion in the anterior pituitary from embryonic period to adulthood. In addition, the expression of kisspeptin was detected in rat LHβ cells (Richard et al., 2008) and coexpression percentage of LHβ and kisspeptin cells increased during development (Ikeda et al., 2017). Therefore, the pituitary may also be a candidate for one of main organs of serum kisspeptin origin. However, *Kiss1* expression in pituitary is mainly regulated in a gonad-independent manner, as kisspeptin expression in pituitary was not found to be different from that in agonadal mice (Ikeda et al., 2017). Although the present study did not evaluate in the expression pattern of pituitary *Kiss1* in OVX and estradiol-treated OVX rats, it may suggest serum kisspeptin is primarily sourced from ovaries, not pituitary. However, *Kiss1* mRNA levels in female mice pituitary increased when puberty began and further increased during postpubertal and adulthood ages (Ikeda et al., 2017). In addition, although the action of kisspeptin on gonadotropin release is primarily mediated *via* hypothalamic GnRH (Shahab et al., 2005), administration of kisspeptin to cultured pituitary cells induced gonadotropin gene expression (Witham et al., 2013) and LH secretion (Gutiérrez-Pascual et al., 2007; Suzuki et al., 2008). These results suggest a special role of pituitary kisspeptin in regulating reproductive function, especially, ovarian differentiation and gonadotrope function during adulthood (Ikeda et al., 2017).

Terao et al. (2004) was the first to report that *Kiss1* was expressed in rat ovaries, which was then confirmed in several studies (Castellano et al., 2006; Laoharatchatathanin et al., 2015; Fernandois et al., 2016). Although different expression patterns were observed among these studies because of age discrepancies and samples obtained having been at different points in the estrous cycle, ovarian *Kiss1* expression has been associated with follicle growth, oocyte maturation, and ovulation (Castellano et al., 2006; Laoharatchatathanin et al., 2015; Fernandois et al., 2016). In addition, ovarian *Kiss1* expression is directly stimulated by LH surge through the LH receptor (Laoharatchatathanin et al., 2015) or by the injection of human chorionic gonadotropin (Castellano et al., 2006), which is blocked by prevention of the preovulatory gonadotropin surge (Castellano et al., 2006). These suggest that ovarian *Kiss1* expression might be regulated by LH (Castellano et al., 2006; Peng et al., 2013; Laoharatchatathanin et al., 2015). To summarize, ovarian kisspeptin might play a role in follicular development and ovulation, which is associated with LH secretion and not hypothalamic kisspeptin. However, hypothalamic *Kiss1* expression increases with puberty, leading to HPG axis activation and increased LH levels. Although whether LH treatment directly affects ovarian *Kiss1* expression has not been elucidated yet, the increase in LH levels is thought to increase ovarian kisspeptin expression. As a result, serum kisspeptin levels increase according to pubertal development. Therefore, although hypothalamic *Kiss1* expression does not directly affect serum kisspeptin, it indirectly affects its pattern during puberty. Thus, although serum kisspeptin could not be a direct biomarker for the onset of puberty or the activation of HPG axis, it can be an indirect indicator for puberty by judging the maturation of reproductive function according to puberty development. Further studies are needed to provide evidence on the association between LH levels and ovarian *Kiss1* expression pre-, peri-, and post-puberty.

In the present study, we evaluated patterns in serum kisspeptin levels as well as *Kiss1* mRNA and protein expression levels in accordance with different developmental stages in various organs. Serum kisspeptin levels increased as puberty progressed, and among the organs studied, the ovaries expressed the highest level of *Kiss1* in a pattern similar to that of serum kisspeptin levels. In addition, serum kisspeptin levels decreased in OVX rats independent of estradiol treatment. Our findings support the conclusion that serum kisspeptin levels mainly depend on *Kiss1* expression in the ovaries, thereby suggesting that the ovaries are the main source of serum kisspeptin; however, these findings need to be verified in future studies with ovarian-specific *Kiss1* knockout rats.

One limitation of our study was that only intracellular expression of *Kiss1* was shown, but the actual amount of ovarian kisspeptin being secreted from the ovaries was not determined. In order to clearly identify the role of serum kisspeptin according to developmental stage and the main source of serum kisspeptin during puberty, studies using *in vitro* ovarian culture will be needed. Furthermore, the exact distinction between AVPV and ARC in the hypothalamus was not confirmed through immunofluorescence staining. Although this study was conducted with anatomical compartments according to the methods of previously published studies, we did not demonstrate that AVPV and ARC were accurately separated. Third, we indicate that serum kisspeptin may be a downstream marker for ovarian activity but does not represent the degree of activation of the HPG axis during puberty; however, since LH stimulates *Kiss1* expression in the ovaries, the serum kisspeptin level might serve as an indirect marker of puberty. Nevertheless, further studies are needed to evaluate the roles of serum kisspeptin and the mechanism of the Kiss1/Kisspeptin system in the development of the reproductive system.

## Data Availability

The original contributions presented in the study are included in the article/Supplementary Material, further inquiries can be directed to the corresponding author.

## References

[B1] Adachi S. Yamada S. Takatsu Y. Matsui H. Kinoshita M. Takase K. (2007). Involvement of anteroventral periventricular metastin/kisspeptin neurons in estrogen positive feedback action on luteinizing hormone release in female rats. J. Reprod. Dev. 53 (2), 367–378. 10.1262/jrd.18146 17213691

[B2] Castellano J. M. GaytanM. Roa J. Vigo E. Navarro V. M. Bellido C. (2006). Expression of KiSS-1 in rat ovary: Putative local regulator of ovulation? Endocrinology 147 (10), 4852–4862. 10.1210/en.2006-0117 16825322

[B3] Clarkson J. Herbison A. E. (2006). Postnatal development of kisspeptin neurons in mouse hypothalamus; sexual dimorphism and projections to gonadotropin-releasing hormone neurons. Endocrinology 147 (12), 5817–5825. 10.1210/en.2006-0787 16959837 PMC6098691

[B4] de Roux N. Genin E. Carel J. C. Matsuda F. Chaussain J. L. Milgrom E. (2003). Hypogonadotropic hypogonadism due to loss of function of the KiSS1-derived peptide receptor GPR54. Proc. Natl. Acad. Sci. U. S. A. 100 (19), 10972–10976. 10.1073/pnas.1834399100 12944565 PMC196911

[B5] Decourt C. Robert V. Anger K. GalibertM. Madinier J. B. Liu X. (2016). A synthetic kisspeptin analog that triggers ovulation and advances puberty. Sci. Rep. 6, 26908. 10.1038/srep26908 27245315 PMC4887910

[B6] Fernandois D. CuevasF. Cruz G. Lara H. E. Paredes A. H. (2016). Kisspeptin is involved in ovarian follicular development during aging in rats. J. Endocrinol. 228 (3), 161–170. 10.1530/JOE-15-0429 26698566

[B7] Gaytán F. Gaytan, M. Castellano J. M. RoMero, M., Roa J. Aparicio B. (2009). KiSS-1 in the mammalian ovary: Distribution of kisspeptin in human and marmoset and alterations in KiSS-1 mRNA levels in a rat model of ovulatory dysfunction. Am. J. Physiol. Endocrinol. Metab. 296 (3), E520–E531. 10.1152/ajpendo.90895.2008 19141682

[B8] Gutiérrez-Pascual E. Martinez-Fuentes A. J. PiniLLa L. Tena-SeMpereM. Malagon M. M. Castano J. P. (2007). Direct pituitary effects of kisspeptin: Activation of gonadotrophs and somatotrophs and stimulation of luteinising hormone and growth hormone secretion. J. Neuroendocrinol. 19 (7), 521–530. 10.1111/j.1365-2826.2007.01558.x 17532794

[B9] Hua W. Luo L. Tian Y. Song M. Liu Y. Cui P. (2014). Analysis of the serum concentrations of kisspeptin and neurokinin B in the geese during reproductive cycle and their localisation in the ovary. Anim. Reprod. Sci. 151, 78–84. 10.1016/j.anireprosci.2014.09.014 25282553

[B10] Ikeda Y. Tagami A. Komada M. Takahashi M. (2017). Expression of kisspeptin in gonadotrope precursors in the mouse pituitary during embryonic and postnatal development and in adulthood. Neuroendocrinology 105 (4), 357–371. 10.1159/000453398 27871073

[B11] Jayasena C. N. Nijher G. M. K. Narayanaswamy S. De Silva A. Abbara A. Ghatei M. A. (2014). Age-dependent elevations in plasma kisspeptin are observed in boys and girls when compared with adults. Ann. Clin. Biochem. 51, 89–96. 10.1177/0004563213485230 23869023

[B12] Kanasaki H. Purwana I. N. Oride A. Mijiddorj T. Sukhbaatar U. Miyazaki K. (2013). Circulating kisspeptin and pituitary adenylate cyclase-activating polypeptide (PACAP) do not correlate with gonadotropin serum levels. Gynecol. Endocrinol. 29 (6), 583–587. 10.3109/09513590.2013.788624 23656386

[B13] Kauffman A. S. Clifton D. K. Steiner R. A. (2007). Emerging ideas about kisspeptin– GPR54 signaling in the neuroendocrine regulation of reproduction. Trends Neurosci. 30, 504–511. 10.1016/j.tins.2007.08.001 17904653

[B14] Kauffman A. S. Gottsch M. L. Roa J. Byquist A. C. Crown A. Clifton D. K. (2007). Sexual differentiation of Kiss1 gene expression in the brain of the rat. Endocrinology 148 (4), 1774–1783. 10.1210/en.2006-1540 17204549

[B15] Khazipov R. Zaynutdinova D. Ogievetsky E. Valeeva G. Mitrukhina O. Manent J. B. (2015). Atlas of the postnatal rat brain in stereotaxic coordinates. Front. Neuroanat. 9, 161. 10.3389/fnana.2015.00161 26778970 PMC4688355

[B16] Laoharatchatathanin T. Terashima R. Yonezawa T. Kurusu S. Kawaminami M. (2015). Augmentation of metastin/kisspeptin mRNA expression by the proestrous luteinizing hormone surge in granulosa cells of rats: Implications for luteinization. Biol. Reprod. 93 (1), 15. 10.1095/biolreprod.115.127902 25995272

[B17] Lee J. H. Miele M. E. Hicks D. J. Phillips K. K. Trent J. M. Weissman B. E. (1996). KiSS-1, a novel human malignant melanoma metastasis-suppressor gene. J. Natl. Cancer Inst. 88 (23), 1731–1737. 10.1093/jnci/88.23.1731 8944003

[B18] Li X.-F. Kinsey-Jones J. S. Cheng Y. Knox A. M. I. Lin Y. Petrou N. A. (2009). Kisspeptin signalling in the hypothalamic arcuate nucleus regulates GnRH pulse generator frequency in the rat. PloS one 4 (12), e8334. 10.1371/journal.pone.0008334 20016824 PMC2789414

[B19] Navarro V. M. FeRnandez-FeRnandez R. Castellano J. M. Roa J. Mayen A. Barreiro M. L. (2004). Advanced vaginal opening and precocious activation of the reproductive axis by KiSS-1 peptide, the endogenous ligand of GPR54. J. Physiol. 561, 379–386. 10.1113/jphysiol.2004.072298 15486019 PMC1665361

[B20] Novaira H. J. Sonko M. L. Hoffman G. Koo Y. Ko C. Wolfe A. (2014). Disrupted kisspeptin signaling in GnRH neurons leads to hypogonadotrophic hypogonadism. Mol. Endocrinol. 28 (2), 225–238. 10.1210/me.2013-1319 24422632 PMC3896637

[B21] Ohtaki T. Shintani Y. Honda S. Matsumoto H. Hori A. Kanehashi K. (2001). Metastasis suppressor gene KiSS-1 encodes peptide ligand of a G-protein-coupled receptor. Nature 411 (6837), 613–617. 10.1038/35079135 11385580

[B22] Ojeda S. R. Urbanski H. F. (1988). “Puberty in the rat,” in The Physiology of reproduction. Editors Knobil E. Neill J. (New York: Raven Press), 1699–1737.

[B23] Peng J. Tang M. Zhang B. P. Zhang P. Zhong T. Zong T. (2013). Kisspeptin stimulates progesterone secretion via the Erk1/2 mitogen-activated protein kinase signaling pathway in rat luteal cells. Fertil. Steril. 99 (5), 1436–1443. 10.1016/j.fertnstert.2012.12.008 23312234

[B24] Pita J. Barrios V. Gavela-Perez T. Martos-Moreno G. A. Munoz-Calvo M. T. Pozo J. (2011). Circulating kisspeptin levels exhibit sexual dimorphism in adults, are increased in obese prepubertal girls and do not suffer modifications in girls with idiopathic central precocious puberty. Peptides 32 (9), 1781–1786. 10.1016/j.peptides.2011.07.016 21827808

[B25] Rao X. Huang X. Zhou Z. Lin X. (2013). An improvement of the 2ˆ(-delta delta CT) method for quantitative real-time polymerase chain reaction data analysis. Biostat. Bioinforma. Biomath. 3 (3), 71–85. 25558171 PMC4280562

[B26] Rhie Y. J. Lee K. H. Eun S. H. Choi B. M. Chae H. W. Kwon A. R. (2011). Serum kisspeptin levels in Korean girls with central precocious puberty. J. Korean Med. Sci. 26 (7), 927–931. 10.3346/jkms.2011.26.7.927 21738347 PMC3124724

[B27] Richard N. Galmiche G. CorvaiSier S. CarAty A. Kottler M. L. (2008). KiSS-1 and GPR54 genes are co-expressed in rat gonadotrophs and differentially regulated *in vivo* by oestradiol and gonadotrophin-releasing hormone. J. Neuroendocrinol. 20 (3), 381–393. 10.1111/j.1365-2826.2008.01653.x 18208554

[B28] Ricu M. A. Ramirez V. D. Paredes A. H. Lara H. E. (2012). Evidence for a celiac ganglion-ovarian kisspeptin neural network in the rat: intraovarian anti-kisspeptin delays vaginal opening and alters estrous cyclicity. Endocrinology 153 (10), 4966–4977. 10.1210/en.2012-1279 22869347

[B29] Roa J. Vigo E. Castellano J. M. Navarro V. M. FeRnandez-FeRnandez R. Casanueva F. F. (2006). Hypothalamic expression of KiSS-1 system and gonadotropin-releasing effects of kisspeptin in different reproductive states of the female Rat. Endocrinology 147 (6), 2864–2878. 10.1210/en.2005-1463 16527840

[B30] Roseweir A. K. Kauffman A. S. Smith J. T. Guerriero K. A. Morgan K. Pielecka-Fortuna J. (2009). Discovery of potent kisspeptin antagonists delineate physiological mechanisms of gonadotropin regulation. J. Neurosci. 29 (12), 3920–3929. 10.1523/JNEUROSCI.5740-08.2009 19321788 PMC3035813

[B31] Semaan S. J. Kauffman A. S. (2015). Daily successive changes in reproductive gene expression and neuronal activation in the brains of pubertal female mice. Mol. Cell. Endocrinol. 401, 84–97. 10.1016/j.mce.2014.11.025 25498961 PMC4312730

[B32] Seminara S. B. Messager S. Chatzidaki E. E. Thresher R. R. Acierno J. S. Shagoury J. K. (2003). The GPR54 gene as a regulator of puberty. N. Engl. J. Med. 349 (17), 1614–1627. 10.1056/NEJMoa035322 14573733

[B33] Shahab M. Mastronardi C. Seminara S. B. Crowley W. F. Ojeda S. R. Plant T. M. (2005). Increased hypothalamic GPR54 signaling: A potential mechanism for initiation of puberty in primates. Proc. Natl. Acad. Sci. U. S. A. 102 (6), 2129–2134. 10.1073/pnas.0409822102 15684075 PMC548549

[B34] Smith J. T. Cunningham M. J. Rissman E. F. Clifton D. K. Steiner R. A. (2005). Regulation of Kiss1 gene expression in the brain of the female mouse. Endocrinology 146 (9), 3686–3692. 10.1210/en.2005-0488 15919741

[B35] Smith J. T. Popa S. M. Clifton D. K. Hoffman G. E. Steiner R. A. (2006). Kiss1 neurons in the forebrain as central processors for generating the preovulatory luteinizing hormone surge. J. Neurosci. 26 (25), 6687–6694. 10.1523/JNEUROSCI.1618-06.2006 16793876 PMC6673844

[B36] Suzuki S. Kadokawa H. Hashizume T. (2008). Direct kisspeptin-10 stimulation on luteinizing hormone secretion from bovine and porcine anterior pituitary cells. Anim. Reprod. Sci. 103 (3-4), 360–365. 10.1016/j.anireprosci.2007.05.016 17604578

[B37] Takase K. UenoYama Y. InoueN. Matsui H. Yamada S. ShiMizuM. (2009). Possible role of oestrogen in pubertal increase of Kiss1/kisspeptin expression in discrete hypothalamic areas of female rats. J. Neuroendocrinol. 21 (6), 527–537. 10.1111/j.1365-2826.2009.01868.x 19500223

[B38] Terao Y. Kumano S. Takatsu Y. Hattori M. Nishimura A. Ohtaki T. (2004). Expression of KiSS-1, a metastasis suppressor gene, in trophoblast giant cells of the rat placenta. Biochim. Biophys. Acta 1678 (2-3), 102–110. 10.1016/j.bbaexp.2004.02.005 15157736

[B39] Topaloglu A. K. Tello J. A. Kotan L. D. Ozbek M. N. Yilmaz M. B. Erdogan S. (2012). Inactivating KISS1 mutation and hypogonadotropic hypogonadism. N. Engl. J. Med. 366 (7), 629–635. 10.1056/NEJMoa1111184 22335740

[B40] Uenoyama Y. Pheng V. Tsukamura H. Maeda K. I. (2016). The roles of kisspeptin revisited: inside and outside the hypothalamus. J. Reprod. Dev. 62 (6), 537–545. 10.1262/jrd.2016-083 27478063 PMC5177970

[B41] Vries L. D. Shtaif B. Phillip M. Gat-Yablonski G. (2009). Kisspeptin serum levels in girls with central precocious puberty. Clin. Endocrinol. 7, 524–528. 10.1111/j.1365-2265.2009.03575.x 19508611

[B42] Witham E. A. Meadows J. D. Hoffmann H. M. Shojaei S. Coss D. Kauffman A. S. (2013). Kisspeptin regulates gonadotropin genes via immediate early gene induction in pituitary gonadotropes. Mol. Endocrinol. 27 (8), 1283–1294. 10.1210/me.2012-1405 23770611 PMC3725344

[B43] Xue J. Song W. Si M. Sun C. Li K. Wang W. (2020). Serum kisspeptin and AMH levels are good references for precocious puberty progression. Int. J. Endocrinol. 2020, 3126309. 10.1155/2020/3126309 33293954 PMC7700058

[B44] Young J. George J. T. Tello J. A. Francou B. Bouligand J. Guiochon-Mantel A. (2013). Kisspeptin restores pulsatile LH secretion in patients with neurokinin B signaling deficiencies: Physiological, pathophysiological and therapeutic implications. Neuroendocrinology 97 (2), 193–202. 10.1159/000336376 22377698 PMC3902960

