# Supplementary figures and images for: Serum kisspeptin levels mainly depend on ovarian expression of Kiss1 mRNA in female rats
Source: Front Physiol. 2022 Nov 15;13:998446. doi: 10.3389/fphys.2022.998446 (PMC9705754; doi:10.3389/fphys.2022.998446)

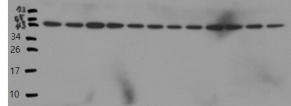

Supplement: Supplementary file 1 [file DataSheet1.ZIP › western supplementary_300DPI(TIF)/F2(B)AVPV,ARC,OVARY_beta-actin.tif]

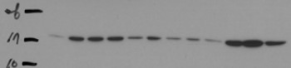

Supplement: Supplementary file 1 [file DataSheet1.ZIP › western supplementary_300DPI(TIF)/F2(B)AVPV,ARC,OVARY_kisspeptin.tif]

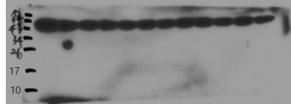

Supplement: Supplementary file 1 [file DataSheet1.ZIP › western supplementary_300DPI(TIF)/F2(B)pitu,adrenal,uterus_beta actin.tif]

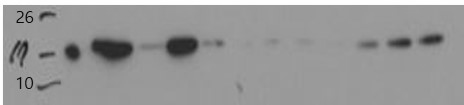

Supplement: Supplementary file 1 [file DataSheet1.ZIP › western supplementary_300DPI(TIF)/F2(B)pitu,adrenal,uterus_kisspeptin.tif]

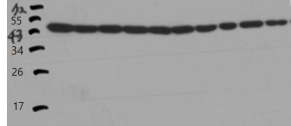

Supplement: Supplementary file 1 [file DataSheet1.ZIP › western supplementary_300DPI(TIF)/F3(C)AVPV_beta-actin.tif]

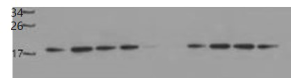

Supplement: Supplementary file 1 [file DataSheet1.ZIP › western supplementary_300DPI(TIF)/F3(C)AVPV_kisspeptin.tif]

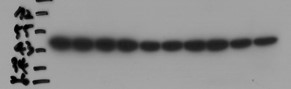

Supplement: Supplementary file 1 [file DataSheet1.ZIP › western supplementary_300DPI(TIF)/F3(D)ARC_beta actin.tif]

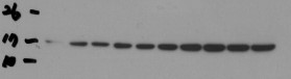

Supplement: Supplementary file 1 [file DataSheet1.ZIP › western supplementary_300DPI(TIF)/F3(D)ARC_kisspeptin.tif]
